# How robust is the association between youth unemployment and later mental health? An analysis of longitudinal data from English schoolchildren

**DOI:** 10.1136/oemed-2021-107473

**Published:** 2021-05-26

**Authors:** Liam Wright, Jenny A Head, Stephen Jivraj

**Affiliations:** 1 Department of Behavioural Science and Health, University College London, London, UK; 2 Department of Epidemiology and Public Health, University College London, London, UK

**Keywords:** epidemiology, mental health, public health

## Abstract

**Background:**

Several studies show that youth unemployment is associated with worse mental health later in life. However, existing studies report results for only one model, or a few models, and use regression adjustment to support causal claims. We use two novel methods to address these gaps in the literature.

**Methods:**

We use data from Next Steps, a cohort study of English schoolchildren who entered the labour market in the aftermath of the 2008–2009 global financial crisis, and measure mental health using the 12-item General Health Questionnaire (GHQ-12) at age 25. We use specification curve analysis and a negative control outcome design (a form of placebo test) to test whether associations between youth unemployment and later GHQ-12 scores are sensitive to model specification or are likely to be confounded by unobserved factors.

**Results:**

We find that the association between unemployment and later GHQ-12 is qualitatively similar across 99.96% of the 120 000 models we run. Statistically significant associations with two placebo outcomes, height and patience, are not present when regression adjustments are made.

**Conclusions:**

There is clear evidence that youth unemployment is related to later mental health, and some evidence that this cannot be easily explained by unobserved confounding.

**Key messages**

**What is already known about this subject?**

- A small literature shows that youth unemployment is associated with worse mental health later in life, but results are based on observational data and reported from one model or a few models.

**What are the new findings?**

- We show that the association is robust to defensible modelling assumptions, such as the measurement of youth unemployment and mental health.
- We show that youth unemployment is not associated with two placebo outcomes, patience and height, when regression adjustments are made.

**How might this impact on policy or clinical practice in the foreseeable future?**

- Youth unemployment is robustly related to later mental health and the association is not easily explained by unobserved confounding. Policymakers should consider targeting interventions at unemployed youth to improve population health.

## Introduction

The pandemic of COVID-19 has caused widespread economic disruption. Unemployment rates have risen across the globe, particularly among young people.^1^ Several studies show that youth unemployment is associated with worse mental health later in life,^2–5^ which, if causal, raises the possibility that the pandemic will have negative impacts on public health for years to come.

The existing literature on the association between youth unemployment and later mental health has relied on observational data. While associations remain after accounting for adolescent mental health (ie, health-related selection into unemployment),^2 4^ other unobserved factors may still confound associations. Existing studies also only present results from one model, or a few models, but a wider universe of models are defensible, including using different operationalisations of mental health and youth unemployment. Results may not be robust to these other choices.

In this paper, we test the robustness of the association between youth unemployment and later mental health using data from a cohort of young people who entered the labour market during the aftermath of the 2008–2009 global financial crisis, a period marked by high youth unemployment rates. We use a novel method—specification curve analysis^6^—to explore whether this association varies substantively across the ‘universe’ of justifiable model specifications. We also test whether youth unemployment is associated with two placebo outcomes, height (ie, vertical stature) and patience (ie, disposition to delay gratification or tolerate present discomfort)—factors that unemployment should have no causal relation with, but which may be related to similar factors confounding the relationship between unemployment and later mental health, such as adolescent health, socioeconomic position and traits such as conscientiousness.^7 8^ Our reasoning is that if we find an association between youth unemployment, height and patience, even after making regression adjustments, this would suggest that associations with later mental health are biased. This is known as a negative control outcome design.^9^

## Methods

### Participants

We use data from Next Steps, a cohort of English schoolchildren recruited at age 13–14 in 2003–04. The cohort was followed annually for 7 years to age 19–20 and surveyed again at age 25. Participants were recruited using a two-stage stratified sampling design with ethnic minority and economically disadvantaged children oversampled. Fifteen thousand seven hundred and seventy individuals were originally recruited with a sample boost of 352 ethnic minority participants added at age 16–17. We use data from all individuals who participated at the age 25 survey (n=7707; 47.8% of total sample).

### Measures

Our primary outcome measure is the 12-item General Health Questionnaire (GHQ-12) at age 25. The GHQ-12 is a screening tool for minor psychiatric morbidity in a general population. Items are scored on a 4-point scale. Higher values indicate worse mental health. Our placebo outcomes are height and patience. Both were measured via self-report at age 25 using a single-item question. We use the placebo outcomes as collected but compare several different procedures to score and combine GHQ items, including Likert (0–1–2–3), Caseness (0–0–1–1) and Corrected (0–0–1–1 for positively phrased items; 0–1–1–1 for negatively phrased items) item scoring and sum scores and factor scores extracted from confirmatory factor analysis.

Employment histories were collected with self-report modules at each wave. We measure youth unemployment using binary variables with different minimum durations, methods of aggregation (cumulative or continuous episodes) and age ranges to define the youth period (16–21, 18–21 and so on). We include several control variables representing factors that predict selection into youth unemployment, such as adolescent mental and physical health, gender, ethnicity, education, socioeconomic background, attitude to schooling, early risk behaviours (eg, cannabis smoking) and locus of control. Further detail on the variables—and their various operationalisations—is provided in the online supplemental information. Note, education was measured subsequent to youth unemployment and risk behaviours and attitude to schooling may not directly cause youth unemployment, instead opening backdoor paths when controlled for,^10^ so we run models adjusting and not adjusting for these factors.

10.1136/oemed-2021-107473.supp1 Supplementary data

### Statistical analysis

Our analysis proceeds in three steps. First, we generate a ‘universe’ of model specifications using combinations and definitions of the different measures that we believe yield theoretically justified tests of an association between youth unemployment and the outcome variables. To reduce computational complexity, our specifications only include linear regression. This amounts to over 15 million model specifications. Second, to make the analysis feasible, we run a random subset of these models—120 000 models using GHQ, and 20 000 each for height and patience—presenting the results graphically to see how the association differs across specifications. Third, we produce inferential statistics using under-the-null-bootstrapping. For each outcome variable, we draw results from 1000 specifications and re-run analyses with 500 bootstrapped samples modified so that the null hypothesis of no association is true by construction. We calculate exact p values by counting the proportion of bootstrap samples that produce more extreme results than the original sample. We use complete case data, given the computational cost and the issues inherent in imputing highly collinear data. We use weights in all specifications to account for attrition from the study.

## Results

**Figure 1:**
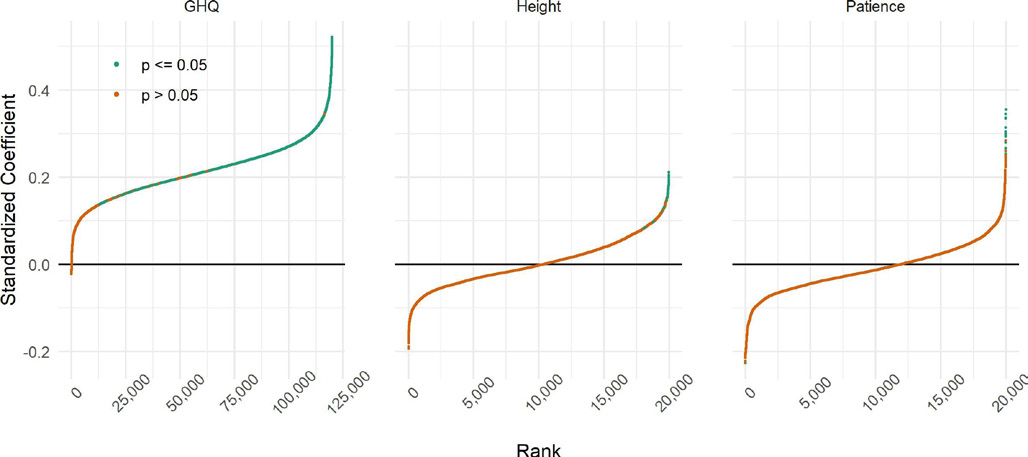
Results of specification curve analysis. Each point represents the result from a single regression. Estimates ranked by effect size. GHQ, General Health Questionnaire.

Using our preferred operationalisations, there was a bivariate association between youth unemployment and GHQ scores (b=0.31, 95% CI=0.18 to 0.43) and patience (b=−0.16, 95% CI=−0.27 to –0.05) but not height (b=0.03, 95% CI=−0.08 to 0.14). Figure 1 presents the range of (standardised) estimates across model specifications. The median effect size for GHQ scores was 0.21 SD. 79.42% of specifications were statistically significant and only 0.04% of specifications predicted better mental health among the youth unemployed. None of the 500 bootstrap samples produced larger median effect sizes, a higher proportion of significant results or larger average z-statistics. 1.93% and 0.1% of specifications reached statistical significance for height and patience, respectively, and the median effect sizes were 0 and −0.01 SD. A small number of specifications reached substantial effect sizes, however. More than half of the bootstrap samples produced more extreme results than the original sample.

Online supplemental figure S1 presents the results of the specification curve analysis for GHQ-12 scores separated by control variables included in regressions. With few exceptions, youth unemployment was associated with later GHQ regardless of the control variables added. Estimated associations were somewhat smaller when educational attainment was excluded from models (median effect size=0.2 SD) and somewhat larger when risk behaviours and attitude to schooling were excluded from models (median effect size=0.25 SD). Online supplemental figure S2 shows the distribution of estimates by minimum duration used to define youth unemployment. Estimates are generally larger when longer minimum durations are used.

A potential issue with the results in this analysis is bias from non-random attrition from the study. Online supplemental figure S3 shows the results of logistic regression models exploring whether attrition was related to the covariates used in the analysis. Unemployed individuals were more likely to drop out of the study, which could generate biases if drop-out was related to mental health. However, there were not clear differences in attrition according to adolescent mental health when adjustment for other covariates was made. As adolescent mental health is a strong predictor of later mental health, this may suggest that drop-out was not related to mental health outcomes.

## Discussion

We find that the association between youth unemployment and later mental health is largely robust to model specification. Estimates differ markedly depending on the exact definitions used to measure variables, however. Associations were robust to controlling for adolescent mental and physical health, suggesting that health-related selection into unemployment does not explain associations. Bivariate associations between youth unemployment and patience were also reduced when including control variables, suggesting that regression adjustment goes some way to reducing unobserved confounding. There was no bivariate association between youth unemployment and height, though height was related to parental socioeconomic class (online supplemental figure S4). This may suggest that selection into unemployment was not as strong as anticipated.

Our results suggest that unemployment could lead to future mental health problems. This is particularly concerning given the current, and expected, increase in youth unemployment during the COVID-19 pandemic. However, as we use observational data, we ultimately cannot rule out that the association is driven by unobserved factors. Other approaches, such as exploiting exogenous causes of youth unemployment would be a beneficial addition to the literature—though identifying such events may be difficult, in practice.

Our finding that associations with later mental health are broadly robust to model specification may increase confidence that results in previous studies are not coincidental or suffer from publication bias, a feature that has been observed in several literatures.^11^ Yet, it should be noted, we have still selected from a wider universe of justifiable specifications—for instance, we did not use other forms of estimation, such as matching or Poisson regression.

## Data Availability

Data from Next Steps are publicly available through the UK Data Service. The code to replicate this analysis is at https://osf.io/y8hfk.
