# Supplementary material for: How robust is the association between youth unemployment and later mental health? An analysis of longitudinal data from English schoolchildren
Source: Occup Environ Med. 2021 May 26;78(8):618–20. doi: 10.1136/oemed-2021-107473 (PMC8292589; doi:10.1136/oemed-2021-107473)
Supplement: Supplementary data [file oemed-2021-107473supp001.pdf]

Supplementary Information

SAMPLE

We used data from Next Steps, a cohort study of 16,122 individuals who were in Year 9 (age 13/14) of secondary school in England in 2003/04. Next Steps was formerly known as the Longitudinal Study of Young People in England (LSYPE). We use the main data files available from the UK Data Service (Centre for Longitudinal Studies, 2018).

There have been eight sweeps of data collection for Next Steps. Cohort members were followed annually for seven years from 2004 (age 13/14) to 2010 (age 19/20) and were interviewed again at age 25 in 2015/16. Cohort members have been interviewed at each sweep, while primary and secondary caregivers were interviewed during the first four years. At each sweep, a small number of "partial interviews" were conducted where the cohort member or their primary or secondary caregivers refused interview.

From Sweeps 1-4, interviews were conducted solely face-to-face. From Sweep 5, interviews were also offered via telephone or online. The majority of interviews from Sweep 5 were conducted this way. Interviews also contained self-completion modules, in which particularly sensitive questions were asked (such as on mental health).

15,770 schoolchildren were initially recruited to the study from an issued sample of 21,000 (response rate 75%). Participants were recruited using a two-stage stratified sampling design with pupils sampled from schools. Maintained (i.e., state) schools were stratified according to the proportion of pupils in receipt of free school meals and the proportion of Year 9 students from minority ethnic groups. Independent schools were stratified according to boarding status, pupil gender, and attainment at GCSE. Deprived schools were oversampled by 50%. Pupil referral units were sampled separately.

Within a given school, pupil sampling probabilities varied according to ethnic group to achieve a minimum sample size of 1,000 in each of six minority ethnic groups (Indian, Pakistani, Bangladeshi, Black African, Black Caribbean, and mixed). Thirty-three pupils were sampled from each school, on average. Only 73%

Wright L, *et al. Occup Environ Med* 2021; 78:618–620. doi: 10.1136/oemed-2021-107473

of sampled schools co-operated with the study (647 of 892) with lower co-operation rates occurring in Inner London and independent schools. In Sweep 4 (age 16/17), a sample boost of 352 individuals from black and minority ethnic (BAME) backgrounds was added from an issued sample of 600 (response rate 59%). This sample was drawn from schools that did not co-operate in Sweep 1. More information on the design of Next Steps can be found in the survey user guides and in a data profile article (Calderwood et al., 2017; Calderwood & Sanchez, 2016; Department for Education, 2011).

From Sweeps 2-7 (ages 14/15 to 19/20), follow-up was only attempted on cohort members who were interviewed in the prior year. Over this period, attrition rates ranged from 8-14%. Just over half (54%) of all participants were interviewed at age 19/20. To maximise sample representativeness, at age 25, all cohort members were approached for follow-up, including those who had not participated in the prior sweep. 50% of the issued sample were interviewed at this age, one quarter (26%) of whom were not interviewed at age 19/20.

MEASURES

Primary Outcome: GHQ-12 @ Age 25

The primary outcome is the 12-item General Health Questionnaire (GHQ-12), which was collected in a self-completion module at the age 25 interview. The GHQ-12 was developed as a screening tool for minor non-psychotic psychiatric morbidity in a general population (Goldberg & Hillier, 1979).

We use three methods for scoring individual items: Likert scoring (0-1-2-3), Caseness (0-0-1-1) scoring and Corrected scoring (0-0-1-1 for positively worded items; 0-1-1-1 for negatively worded items). It is argued that Corrected scoring better captures chronic mental health problems (Peasgood et al., 2014). We use two ways of combining the items: sum scores and confirmatory factor analysis (CFA). We extract factor scores using Rodrigo et al.'s (2019) CFA model which models item responses with three latent factors: a single factor capturing psychological distress upon which every item is loaded and two separate factors capturing method effects, with negative and positive-worded questions loaded onto separate factors. We estimate CFA models in lavaan version 0.6-6 (Rosseel, 2012) using the Diagonally Weighted Least Squares estimator (DWLS) given that items are skewed and unlikely to be jointly normally distributed (Rodrigo et

al., 2019). The Rodrigo et al. (2019) model has superior fit statistics to other CFA models suggested in the literature. Our primary measure of GHQ is the Likert sum score.

Negative Control "Placebo" Outcomes: Patience and Height @ Age 25

We use two negative control outcomes to test whether the association between youth unemployment and mental health is likely to be driven by confounding: patience and height, both measured at age 25. Patience is measured with a single interviewer-administered question: "[on] a scale of 0-10, where 0 is very impatient and 10 is very patient, how patient would you say you are?" We analyse this as a continuous variable. Height was collected using via interview question, rather than by direct physiological measurement. Participants were able to respond with metric (metres) or imperial (feet and inches) measurements. We use height as a continuous variable (metres).

Height and patience show bivariate associations with parental socio-economic class and education level, respectively (Figures S4 and S5).

Exposure: Youth Unemployment

Our primary measure of youth unemployment is 6+ months continuous unemployment between October 2008 and May 2010 (approximately age 18-20). This period is the first twenty months after the summer holidays following the normative end of further education (i.e., A-Levels). The period overlaps with the end of the 2008/09 Great Recession and the beginning of its aftermath, in which youth unemployment rates rose to over 18% in the UK.

We choose this period as it overlaps with first entry in to the labour market for many of the individuals in this cohort. We begin after the summer holidays to exclude episodes of unemployment from individuals who intend to go back to education. Youth worklessness rates increase substantially over academic holidays (Furlong, 2006), but are less likely to have long term impacts on labour market success and wellbeing, given that summer jobs at that age may typically be short-term, unrelated to future career aims, less likely to be looked on favourably by prospective employers (Baert et al., 2016), and students are likely to have access to other identities and activities that may protect against negative harms of unemployment (Creed & Evans, 2002; Jahoda, 1982; Paul & Batinic, 2010). We use a cut-off of 6+ months for consistency with other youth unemployment scarring studies (Hammarström & Janlert, 2002; Strandh et al., 2014), and with government statistics (e.g. ONS, 2019b) and policies, such as Labour's New Deal for Young People, which use six months unemployment to define program eligibility (Myck, 2002).

Wright L, *et al. Occup Environ Med* 2021; 78:618–620. doi: 10.1136/oemed-2021-107473

These choices are somewhat arbitrary. Alternative definitions could have differed on the minimum durations, used cumulative rather the continuous unemployment, have used a different time frame, including using other start and end years or measuring unemployment in a set number of years after leaving full time education (FTE). Theory does not clearly dictate that effects should be observed for only some of these – though, longer unemployment durations are expected to have greater long-term effects. We create 192 binary youth unemployment indicators from combinations of the following:

- Minimum duration: 3, 6, 9 and 12 months
- Time range: 1-4 years
    - Start dates October 2006-2012 and end dates September 2007-2013
    - Years after first leaving full time education.
- Statistic: cumulative or continuous duration

We define a person as having left full time education if they do not return to full time education within 12 months. We use binary, rather than continuous variables, to aid comparison across specifications and because the effect of unemployment may be non-linear in length.

Note, the comparator group differs according to the definition of unemployment used. When using the period after leaving FTE, we use data from only those who have left education. (To focus on long-term associations, we use data from individuals where the unemployment measurement period ends two or more years before the age 25 interview.) When using set date ranges, we compare those who were unemployed during the period against those who were not, including those still in education.

Covariates

We use several control variables to attempt to account for non-random selection into unemployment. To account for mental health-related selection, we use scores from the GHQ-12 at ages 14/15 and 16/17. We use the Caseness or Corrected score at age 14/15 as participants were able to respond "don't know" to each item at this interview (we assume this reflects not experiencing the symptom). We use the Caseness, Corrected, or Likert scores at age 16/17 (range 0-36). Again, we combine items using either sum scores or by extracting factor scores using the Rodrigo et al. (2019) model. Again, this performs better than other popular CFA models.

To account for physical health-related selection, we control for self-rated health at ages 14/15 and 16/17 (categories: very good, fairly good, not very good, not good at all) and for whether the participant had a disability at ages 13/4 or 14/15 (categories: no disability; disability, but schooling unaffected; disabled and schooling affected).

To account for differences in human capital, we control for highest academic qualification at age 25 measured using the National Vocational Qualification (NVQ) scale (six categories: NVQ levels 1-5, no qualifications). Education data is not publicly available for ages prior to this. To capture major demographic differences, we include demographic variables for gender and ethnicity (categories: White, mixed, Indian, Pakistani, Bangladeshi, Black African, Black Caribbean, other).

To account for differences in socio-economic position (SEP), we include variables for the participant's family social class (categories: higher, intermediate, routine/manual, long-term unemployed) and highest parental education (categories: degree, other higher education, A-Level, GCSE A-C, other/none). As an alternative measure we measure socio-economic background by extracting a latent SEP factor from a multiple correspondence analysis including family social class and parental education variables as well as housing tenure at age 13/14 (categories: owned without mortgage; owned with mortgage; council rent; private rent/other).

To capture neighbourhood deprivation, we use the Index of Multiple Deprivation 2004 (IMD) at age 14/15. The IMD is created by the UK Government and captures local area deprivation across seven dimensions (income, employment, health, education, barriers to housing and services, living environment and crime). It is measured at the lower layer super output area (LSOA) level.[1] We use either continuous IMD or IMD quintiles in the SCA to capture possible non-linear effects.

We also include variables for positive attitude to school (response to 12-item measure, range 0-48), risk behaviours (summed response to 8-item measure on anti-social behaviour, alcohol, smoking and drug use in previous 12 months, range 0-8), and bullying victimisation (number of sweeps reported being bullied in prior 12 months, age 13/14 – 15/16, range 0-3). We include these as proxy measures of social adjustment and non-cognitive skills (e.g., impulsivity, conscientiousness) which may predict labour market difficulties and mental health.[2] School attitude and risk behaviours are measured each year from age 13/14 to 15/16. It is unclear at which age these variables may most strongly reflect non-cognitive skills. In the SCA, we loop over (a) school attitude and risk behaviour variables measured at different ages and (b) school attitude and risk behaviour factors extracted from separate exploratory factor analyses (EFA, principal factor) using the school attitude and risk behaviours measures at each age (Eigenvalues 1.83 and 1.91, respectively). The

[1] LSOAs comprise approximately 500 households, on average (mean population 1,500).
[2] Bullying victimization is related to poorer socio-economic and mental health outcomes in adulthood (Brown & Taylor, 2008; Varhama & Björkqvist, 2005; Wolke et al., 2013), though these studies are (understandably) based on observational data.

individual items upon which the school attitude and risk behaviour measures are based are displayed in Tables S1 and S2.

Table S1: Attitude to School. Each item measured on five point scale: strongly, agree, disagree, strongly disagree, don't know. Summed attitude to school measure sum responses using don't know as middle category and reverse coding negative worded items, so higher total score indicate more positive attitude to school.

Item
1. I am happy when I am at school
2. School is a waste of time for me
3. School work is worth doing
4. Most of the time I don't want to go to school
5. People think my school is a good school
6. On the whole I like being at school
7. I work as hard as I can in school
8. In lessons, I often count the minutes till it ends
9. I am bored in lessons
10. The work I do is a waste of time
11. The work I do in lessons is a waste of time
12. I get good marks for my work

Table S2: Risk Behaviours

Item
Played truant in last 12 months
Ever smoked cigarettes
Frequency of smoking cigarattes
Whether ever had proper alcoholic drink
Whether had alcoholic drink in last 12 months
Frequency of having alcoholic drink in last 12 months

Wright L, *et al. Occup Environ Med* 2021; 78:618–620. doi: 10.1136/oemed-2021-107473

Whether ever tried cannabis
Whether ever graffitied on walls
Whether ever vandalised public property
Whether ever shoplifted
Whether ever taken part in fighting or public disturbance

We also include locus of control (LOC) in models. LOC was measured at age 14/15 with participants asked for their level of agreement (strongly agree, agree, disagree, strongly disagree, don't know) with six separate statements. Three of the statements were worded to reflect an internal LOC ("if someone is not a success in life, it is usually their own fault"; "I can pretty much decide what will happen in my life"; "if you work hard at something you'll usually succeed") and three worded to reflect an external LOC ("even if I do well at school, I'll have a hard time getting the right type of job", "people like me don't have much of a chance in life", "how well you get on in this world is mostly a matter of luck"). We place responses onto a five-point scale, centred around "don't know", with external-worded items reverse coded so higher scores indicate less external LOC.[3]

There is no agreed way of combining items to operationalise LOC, both in the Next Steps data specifically 13.and in other large-scale survey datasets which measure LOC (Buddelmeyer & Powdthavee, 2016; Caliendo et al., 2015; Cobb-Clark et al., 2014; Piatek & Pinger, 2016). Researchers using Next Steps data have used different subsets of the LOC items (cf. Crawford et al., 2011; Mendolia & Walker, 2015; Ng-Knight & Schoon, 2017; Wijedasa, 2017),[4] and combined them in several ways, including summing Likert responses (Crawford et al., 2011; Ng-Knight & Schoon, 2017) or extracting latent LOC scores using principal component analysis (Wijedasa, 2017), EFA (Mendolia & Walker, 2015), or CFA (Gladwell et al., 2016).

[3] CFAs perform similar well if don't know is used or treated as a missing value and extracted factors explain similar proportions of the variation in GHQ-12 Likert scores at age 25. The don't know response is used by 3,513 individuals, so discarded this information reduces complete case sample sizes substantially.

[4] Wijedasa (2017) includes another item, "[w]orking hard at school now will help me get on later in life", their LOC measure, while Crawford et al. (2011) include this plus another item, "[d]oing well at school means a lot to me". We do not include these items as they arguably capture opinions about the value of secondary education rather than LOC. Ng-Knight & Schoon (2017) and Gladwell et al. (2016) use the three internal-worded items introduced above, only.

Though internal and external-worded items were originally conceptualized as tapping opposite ends of a single spectrum (Rotter, 1966), a number of exploratory factor analyses have found internal and external worded items load onto separate factors (Caliendo et al., 2015; Cobb-Clark & Schurer, 2013; Piatek & Pinger, 2016), including an analysis of Next Steps (Mendolia & Walker, 2015). In the Next Steps data, a CFA with two correlated latent factors has superior fit statistics to a single factor CFA model (Table S3). The correlation between the latent factors is just 0.4.

Table S3: Fit Statistics from Confirmatory Factor Analyses of Locus of Control Items

Model	RMSEA (95% CI)	CFI
One Factor	0.073 (0.069, 0.078)	0.818
Two Factor	0.044 (0.039, 0.05)	0.941
Hankins (2008)	0.034 (0.028, 0.04)	0.975

However, an alternative explanation is that responses reflect method effects. A single factor CFA with internal-worded items allowed to covary (i.e akin to Hankins (2008) GHQ model) has superior fit statistics to the two-factor solution (Table S3). Given the possibility of method effects, the widespread use of only the first factor from EFA (Cobb-Clark, 2015), and the appeal of understanding locus of control as a singular construct (Rotter, 1975), our first measure of LOC is calculated by extracting a single factor using the Hankins (2008) CFA model (DWLS estimator).

We use several alternate operationalizations of LOC. First, we use the sum Likert score of the six items (Buddelmeyer & Powdthavee, 2016; Elkins et al., 2017). Second, following Caliendo et al. (2015) we take seriously the possibility that items reflect two separate, correlated constructs by (a) using sum Likert scores for internal and external worded items separately, (b) using the first two (standardized) factors from the two factor CFA solution, (c) classifying an individual as “internal” if they score above the median on both factors from the CFA, as “external” if they score below the median on both factors, and “neither” otherwise, and (d) recreating this categorical variable but using 25th/75th percentile cut-off points instead.

Statistical Analysis

We specify three separate models to estimate the association between youth unemployment and GHQ-12 scores and placebo outcomes. Model 1 adds all control variables defined above. Model 2 repeats Model 1 but removes education as a control variable as this is measured at age 25 and could feasibly mediate the association. Model 3 repeats Model 1 but does not include controls for risk behaviours, attitude to school,

and bullying victimization. The pathways between these factors and youth unemployment may already be accounted for with other variables in the model (e.g., educational attainment and adolescent mental health). Including these variables could induce collider bias, if adjusting for these variables opens backdoor paths between youth unemployment and mental health (VanderWeele, 2019).

The SCA includes the combinations of models 1, 2, and using different outcome measures, definitions of youth unemployment, measurement of youth unemployment, and definitions of each control variable. Where the specification uses youth unemployment spells beginning before 2008 or uses FTE to index the youth unemployment period, covariates measured at age 16/17 are removed from models as these may mediate effects. This leaves 17.5 million model specifications. To make the SCA computationally feasible, we run a random subset of 160,000 models, 20,000 for each specific outcome measure (120,000 for GHQ). WE standardize outcome variables to aid comparison across the different models. Survey weights are used in all regressions, but we only use complete case analysis due to high multicollinearity between alternative variables in the SCA and computational difficulties of analyzing multiple datasets.

To formally test whether the SCA models are consistent with an association between youth unemployment and later mental health overall, we produce inferential statistics using the under-the-null bootstrapping procedure suggested by Simonsohn et al. (2019). The procedure runs as follows.

- For each specification, a new dependent variable is created which removes the association between youth unemployment and age 25 GHQ-12 scores estimated in that specification.
- For instance, if GHQ scores were 1 unit higher among those who were youth unemployed, the new dependent variable would be equal to the observed score minus 1 if the individual was unemployed.
- Bootstrap samples are taken from the dataset (with replacement). Each specification is repeated in each bootstrap sample using the alternative dependent variable. In the bootstrap samples, there should be no association between youth unemployment and later mental health by construction.
- For each bootstrap sample, three summary statistics are calculated.
    a) The proportion of specification that are statistically significant ($p < 0.05$) in the expected direction (i.e., showing higher GHQ scores among those who were unemployed).
    b) The median effect size across specifications.
    c) The average z-statistic for the main effect.

The statistics are then compared against corresponding statistics from the main SCA analysis and summed across bootstraps to calculate three exact p-values giving the proportion of bootstraps with more extreme

results (in the expected direction) than the main SCA analysis. If there is strong evidence of an association across specifications, then few bootstraps should produce more extreme effect sizes or a higher number of significant results. Because of the computational cost, we produce inferential statistics using 500 bootstrap samples on a random subset of 1,000 models from the 120,000 included in the SCA. We also repeat this exercise using the two negative control outcome measures.

RESULTS

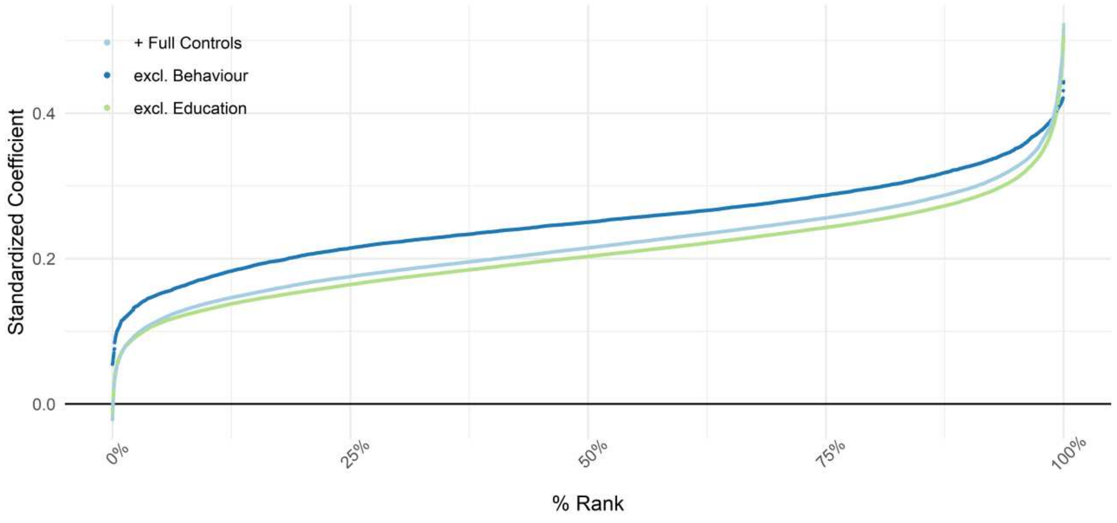

Figure S1: Results of GHQ-12 Specification Curve Analysis by control variables added to regression model. Each point represents the result from a single regression. Estimates ranked by effect size.

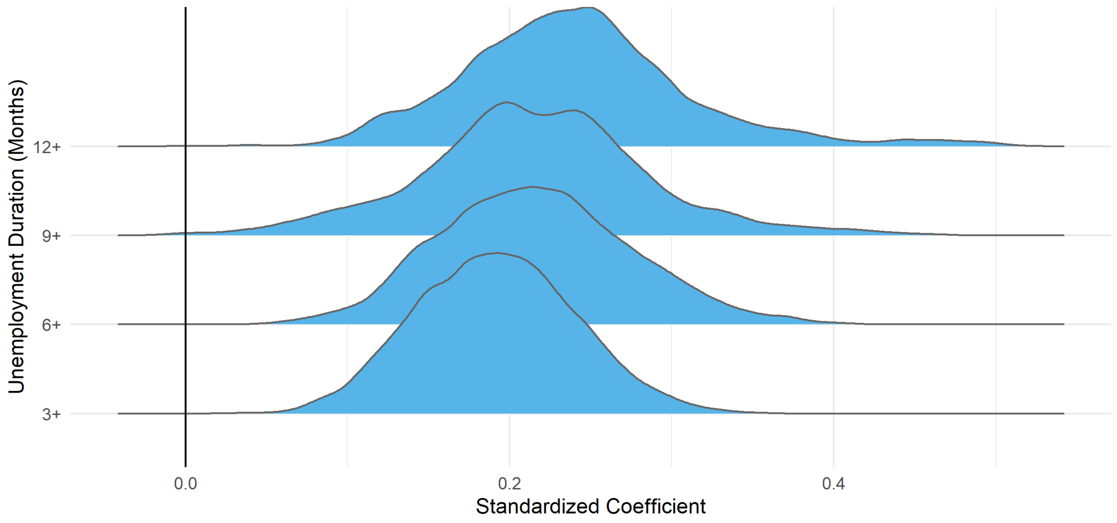

Figure S2: Distribution of GHQ-12 Specification Curve Analysis estimates by minimum duration used to define youth unemployment.

Wright L, *et al. Occup Environ Med* 2021; 78:618–620. doi: 10.1136/oemed-2021-107473

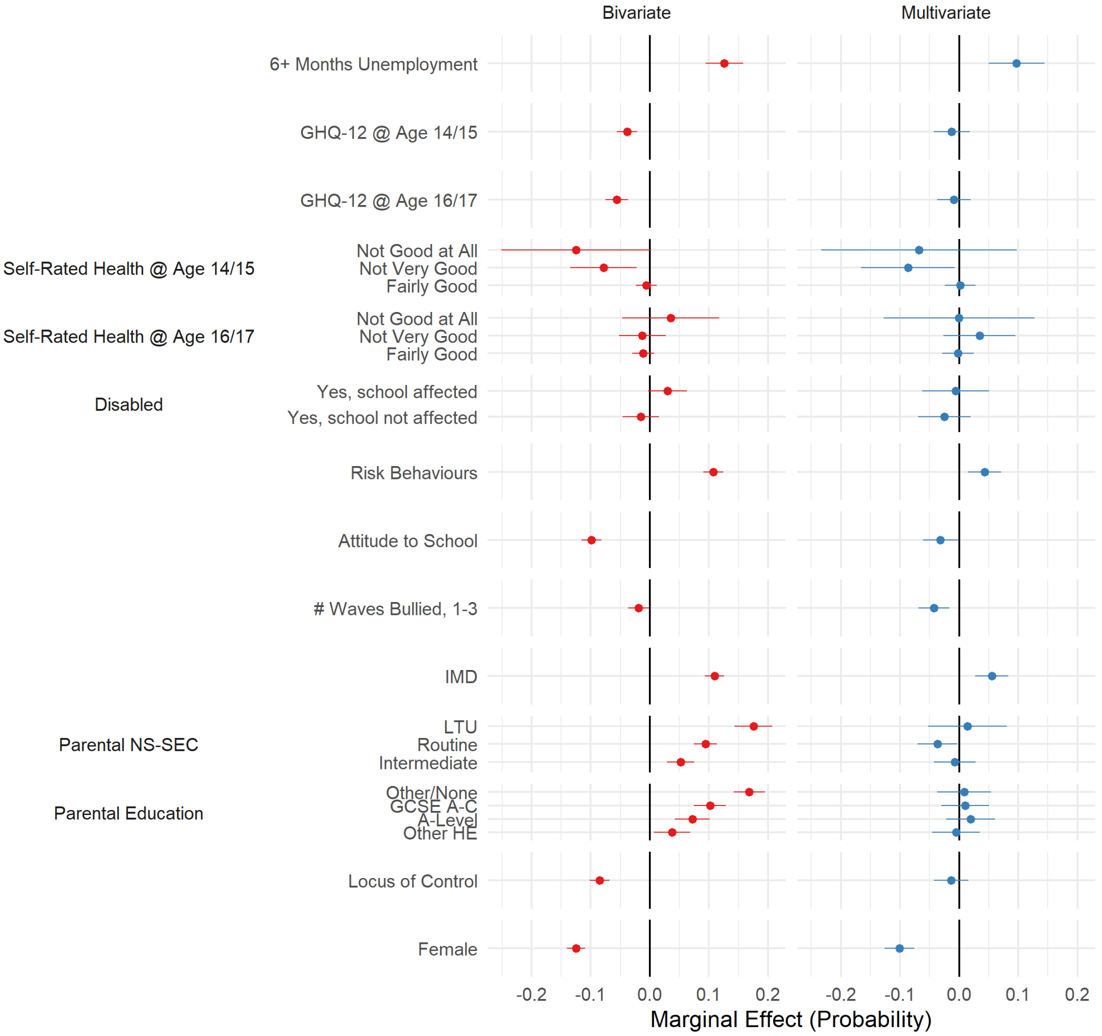

Figure S3: Association between dropping out of Next Steps by age 25 interview and variables used in the SCA (primary definitions used here), derived from logistic regression models. Associations presented at average marginal effects. Multivariate regressions (right panel) include all variables listed in figure as independent variables. Note, complete case data used in this analysis, so the sample differs from the bivariate regressions (left panel) depending on predictor variables used.

Wright L, *et al. Occup Environ Med* 2021; 78:618–620. doi: 10.1136/oemed-2021-107473

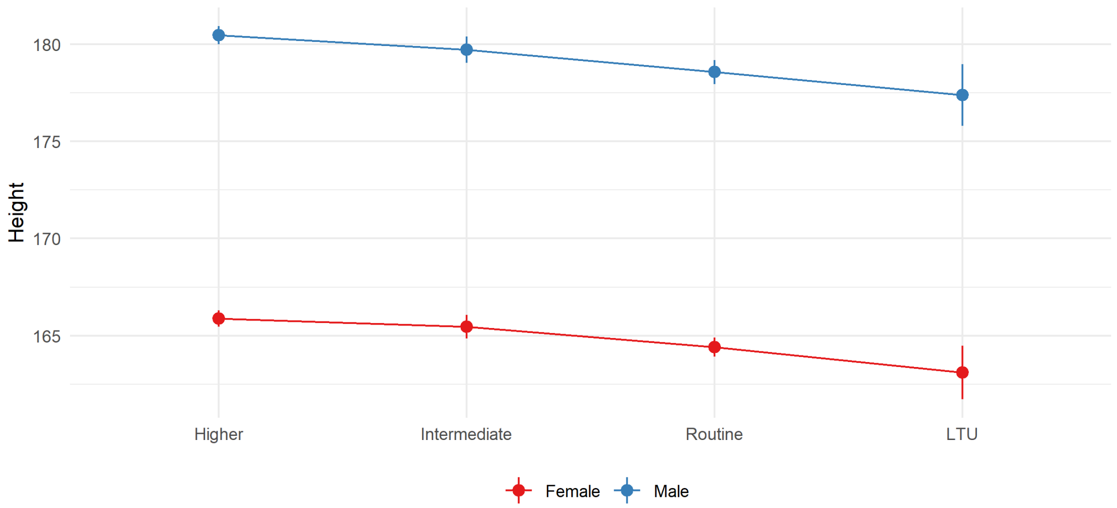

Figure S4: Height at age 25 by parental socio-economic class at age 13/14 and gender. Derived from weighted linear regression model.

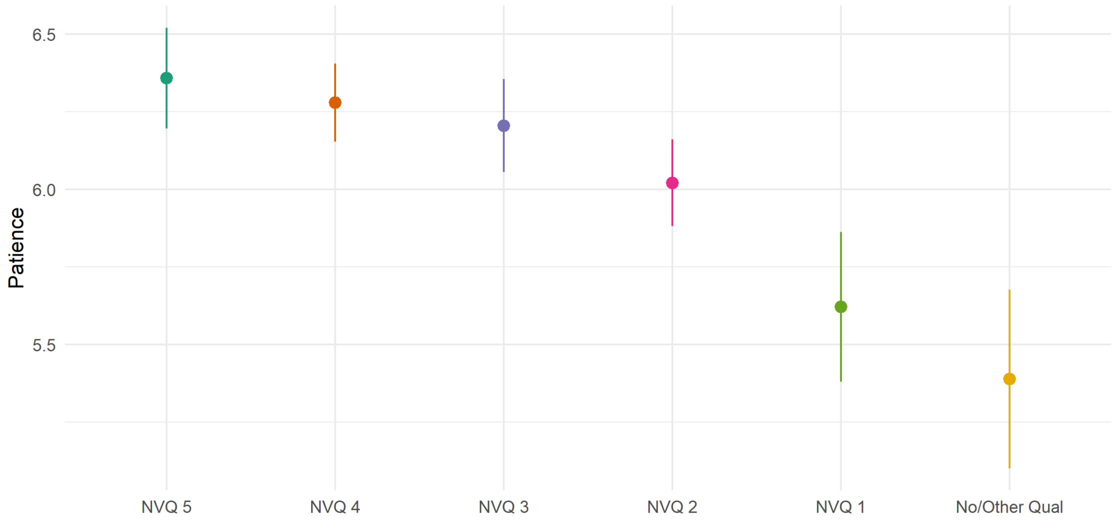

Figure S5: Patience at age 25 by educational attainment. Derived from weighted linear regression model.

Wright L, *et al. Occup Environ Med* 2021; 78:618–620. doi: 10.1136/oemed-2021-107473

REFERENCES

Baert, S., Rotsaert, O., Verhaest, D., & Omey, E. (2016). Student Employment and Later Labour Market Success: No Evidence for Higher Employment Chances. Kyklos, 69(3), 401–425. https://doi.org/10.1111/kykl.12115

Brown, S., & Taylor, K. (2008). Bullying, education and earnings: Evidence from the National Child Development Study. Economics of Education Review, 27(4), 387–401. https://doi.org/10.1016/j.econedurev.2007.03.003

Buddelmeyer, H., & Powdthavee, N. (2016). Can having internal locus of control insure against negative shocks? Psychological evidence from panel data. Journal of Economic Behavior & Organization, 122, 88–109. https://doi.org/10.1016/j.jebo.2015.11.014

Calderwood, L., Peycheva, D., Henderson, M., Mostafa, T., & Rihal, S. (2017). Next Steps: Sweep 8-Age 25 Survey User Guide (First Edition) (p. 37). http://doi.org/10.5255/UKDA-SN-5545-6

Calderwood, L., & Sanchez, C. (2016). Next Steps (formerly known as the Longitudinal Study of Young People in England). Open Health Data, 4, e2. https://doi.org/10.5334/ohd.16

Caliendo, M., Cobb-Clark, D. A., & Uhlendorff, A. (2015). Locus of control and job search strategies. Review of Economics and Statistics, 97(1), 88–103. https://doi.org/10.1162/REST_a_00459

Centre for Longitudinal Studies. (2018). Next Steps: Sweeps 1-8, 2004-2016 (SN: 5545) (14th ed.). UK Data Service. http://doi.org/10.5255/UKDA-SN-5545-6

Cobb-Clark, D. A. (2015). Locus of control and the labor market. IZA Journal of Labor Economics, 4(1), 3. https://doi.org/10.1186/s40172-014-0017-x

Cobb-Clark, D. A., Kassenboehmer, S. C., & Schurer, S. (2014). Healthy habits: The connection between diet, exercise, and locus of control. Journal of Economic Behavior & Organization, 98, 1–28. https://doi.org/10.1016/j.jebo.2013.10.011

Cobb-Clark, D. A., & Schurer, S. (2013). Two economists' musings on the stability of locus of control. Economic Journal, 123(570). https://doi.org/10.1111/ecoj.12069

Crawford, C., Dearden, L., & Greaves, E. (2011). Does when you are born matter?The impact ofmonthof birth onchildren's cognitive and non-cognitive skills in England. Institue for Fiscal Studies. https://www.ifs.org.uk/bns/bn122.pdf

Creed, P. A., & Evans, B. M. (2002). Personality, well-being and deprivation theory. Personality and Individual Differences, 33(7), 1045–1054. https://doi.org/10.1016/S0191-8869(01)00210-0

Department for Education. (2011). LSYPE User Guide to the Datasets: Wave 1 to Wave 7 (p. 103). http://doi.org/10.5255/UKDA-SN-5545-6

Elkins, R. K., Kassenboehmer, S. C., & Schurer, S. (2017). The stability of personality traits in adolescence and young adulthood. Journal of Economic Psychology, 60, 37–52. https://doi.org/10.1016/j.joep.2016.12.005

Furlong, A. (2006). Not a very NEET solution. Work, Employment and Society, 20(3), 553–569. https://doi.org/10.1177/0950017006067001

Gladwell, D., Popli, G., & Tsuchiya, A. (2016). A Dynamic Analysis of Skill Formation and NEET status. https://cemapre.iseg.ulisboa.pt/educonf/4e3/files/Papers/Popli.pdf

Goldberg, D. P., & Hillier, V. F. (1979). A scaled version of the General Health Questionnaire. Psychological Medicine, 9(1), 139–145. https://doi.org/10.1017/S0033291700021644

Hammarström, A., & Janlert, U. (2002). Early unemployment can contribute to adult health problems: Results from a longitudinal study of school leavers. Journal of Epidemiology & Community Health, 56(8), 624–630. https://doi.org/10.1136/jech.56.8.624

Hankins, M. (2008). The factor structure of the twelve item General Health Questionnaire (GHQ-12): The result of negative phrasing? Clinical Practice and Epidemiology in Mental Health, 4(1), 10. https://doi.org/10.1186/1745-0179-4-10

Jahoda, M. (1982). Employment and Unemployment: A Social-Psychological Analysis. Cambridge University Press.

Mendolia, S., & Walker, I. (2015). Youth unemployment and personality traits. IZA Journal of Labor Economics, 4(1). https://doi.org/10.1186/s40172-015-0035-3

Myck, M. (2002). How the New Deal works. Institue for Fiscal Studies. https://www.ifs.org.uk/economic_review/fp193.pdf

Ng-Knight, T., & Schoon, I. (2017). Can Locus of Control Compensate for Socioeconomic Adversity in the Transition from School to Work? Journal of Youth and Adolescence, 46(10), 2114–2128. https://doi.org/10.1007/s10964-017-0720-6

ONS. (2019a). Dataset: Labour market statistics time series. https://www.ons.gov.uk/employmentandlabourmarket/peopleinwork/employmentandemployeetypes/datasets/labourmarketstatistics

ONS. (2019b). Employment in the UK: June 2019. https://www.ons.gov.uk/employmentandlabourmarket/peopleinwork/employmentandemployeetypes/bulletins/employmentintheuk/latest

Paul, K. I., & Batinic, B. (2010). The need for work: Jahoda's latent functions of employment in a representative sample of the German population: THE NEED FOR WORK: LATENT FUNCTIONS OF EMPLOYMENT. Journal of Organizational Behavior, 31(1), 45–64. https://doi.org/10.1002/job.622

Peasgood, T., Brazier, J., Mukuria, C., & Rowen, D. (2014). A conceptual comparison of well-being measures used in the UK. Policy Research Unit in Economic Evaluation of Health & Care Interventions (EEPRU). http://www.eepru.org.uk/article/a-conceptual-comparison-of-well-being-measures-used-in-the-uk/

Piatek, R., & Pinger, P. (2016). Maintaining (Locus of) Control? Data Combination for the Identification and Inference of Factor Structure Models. Journal of Applied Econometrics, 31(4), 734–755. https://doi.org/10.1002/jae.2456

Rodrigo, M. F., Molina, J. G., Losilla, J.-M., Vives, J., & Tomás, J. M. (2019). Method effects associated with negatively and positively worded items on the 12-item General Health Questionnaire (GHQ-12): Results from a cross-sectional survey with a representative sample of Catalonian workers. BMJ Open, 9(11), e031859. https://doi.org/10.1136/bmjopen-2019-031859

Rosseel, Y. (2012). lavaan: An R Package for Structural Equation Modeling. Journal of Statistical Software, 48(2). https://doi.org/10.18637/jss.v048.i02

Rotter, J. B. (1966). Generalized expectancies for internal versus external control of reinforcement. Psychological Monographs, 80(1), 1–28. https://doi.org/10.1037/h0092976

Rotter, J. B. (1975). Some problems and misconceptions related to the construct of internal versus external control of reinforcement. Journal of Consulting and Clinical Psychology, 43(1), 56–67. https://doi.org/10.1037/h0076301

Simonsohn, U., Simmons, J. P., & Nelson, L. D. (2019). Specification Curve: Descriptive and Inferential Statistics on All Reasonable Specifications. SSRN Working Paper. https://doi.org/10.2139/ssrn.2694998

Strandh, M., Winefield, A., Nilsson, K., & Hammarström, A. (2014). Unemployment and mental health scarring during the life course. European Journal of Public Health, 24(3), 440–445. https://doi.org/10.1093/eurpub/cku005

VanderWeele, T. J. (2019). Principles of confounder selection. European Journal of Epidemiology, 34(3), 211–219. https://doi.org/10.1007/s10654-019-00494-6

Varhama, L. M., & Björkqvist, K. (2005). Relation between School Bullying during Adolescence and Subsequent Long-Term Unemployment in Adulthood in a Finnish Sample. Psychological Reports, 96(2), 269–272. https://doi.org/10.2466/pr0.96.2.269-272

Wijedasa, D. (2017). 'People like me don't have much of a chance in life': Comparing the locus of control of young people in foster care with that of adoptees, children from disadvantaged backgrounds and children in the general population. Adoption & Fostering, 41(1), 5–19. https://doi.org/10.1177/0308575916684299

Wolke, D., Copeland, W. E., Angold, A., & Costello, E. J. (2013). Impact of Bullying in Childhood on Adult Health, Wealth, Crime, and Social Outcomes. Psychological Science, 24(10), 1958–1970. https://doi.org/10.1177/0956797613481608
